# Putative Fusion-Associated Small Transmembrane (FAST) Proteins Encoded by Viruses of *Pistolviridae*, Order *Ghabrivirales*, Identified from In Silico Analyses

**DOI:** 10.3390/v18020193

**Published:** 2026-02-01

**Authors:** Racheal Amono, Turhan Markussen, Øystein Evensen, Aase B. Mikalsen

**Affiliations:** Faculty of Veterinary Medicine, Norwegian University of Life Sciences, 1433 Ås, Norway; racheal.amono@nmbu.no (R.A.); turhan.markussen@nmbu.no (T.M.); oystein.evensen@nmbu.no (Ø.E.)

**Keywords:** FAST-proteins, fusion protein, pistolviruses

## Abstract

Fusion-associated small transmembrane (FAST) proteins are viral nonstructural proteins known to be encoded by specific members of the *Spinareoviridae*, specifically within the *Aquareovirus* and *Orthoreovirus* genera. These proteins specialize in mediating cell–cell fusion, leading to syncytia. Unlike enveloped viruses, naked viruses do not rely on fusion proteins for cell entry; however, such proteins may facilitate viral spread between cells. Although not essential for virus replication, FAST proteins have been shown to enhance viral replication, particularly during the early stages of infection. More recently, proteins with characteristics resembling FAST proteins have been identified in a broader range of viruses, including several rotavirus species within the family *Sedoreoviridae*, and, unexpectedly, in some enveloped viruses within the *Coronaviridae* family. Here, we present protein sequence analyses suggesting that viruses of the recently established virus family *Pistolviridae* (order *Ghabrivirales*) also encode proteins with similarity to FAST proteins. Pistolviruses are small double-stranded RNA viruses that infect piscine species, and were initially referred to as “toti-like” viruses due to genomic similarities with members of the former *Totiviridae*, which infect single-celled organisms. The putative FAST proteins of the pistolviruses may be expressed either from small, distinct open reading frames or suggested to be produced as cleavage products derived from polyproteins.

## 1. Introduction

*Pistolviridae* is a new virus family recently added to the order *Ghabrivirales*, suborder *Betatotivirinae* [1,2]. Pistolviruses (derived from Piscine toti-like viruses) include viruses with a double-stranded RNA genome of 6.5–6.8 kb in size, encapsidated in spherical particles of approximately 50 nm in diameter [1,3]. At present, the virus family includes piscine myocarditis virus (PMCV), golden shiner toti-like virus 1 (GSTLV-1), Cyclopterus lumpus toti-like virus (CLuTLV) and common carp toti-like virus 1 (CCTLV-1), hosted by Atlantic salmon (*Salmo salar*), golden shiner (*Notemigonus crysoleucas*), common carp (*Cyprinus carpio*) and lumpsucker (*Cyclopterus lumpus*), respectively. Recently, a virus designated as sea bass toti-like virus (SBTLV) was identified in European sea bass (*Dicentrarchus labrax*) [4]. SBTLV exhibits characteristics consistent with members of the family *Pistolviridae* and should be considered for inclusion in this family in future taxonomic updates.

PMCV is the only virus among the five assigned and putative members of *Pistolviridae* that causes disease in its host, aquacultured Atlantic salmon. PMCV may be seen as a systemic infection, however only the heart is described with lesions in the tissue, characterized as myocarditis [5,6]. The disease, named cardiomyopathy syndrome (CMS), is usually found in large fish near slaughter and is a severe welfare problem for the fish and an economic burden to the producers.

Consistent with most members of the *Ghabrivirales* order, pistolviruses possess a non-segmented genome that encodes a single capsid protein (ORF1) and an RNA-dependent RNA polymerase (RdRp; ORF2). However, pistolviruses are unique in harboring additional coding sequences at the 3′ end of the genome. In PMCV, GSTLV-1, and CCTLV, this region comprises a third open reading frame (ORF3) ranging from 0.9 to 1.1 kb. CLuTLV and SBTLV contain a comparable number of additional nucleotides at the 3′ end; however, in these two viruses, these are distributed across multiple smaller ORFs, ORF3 to ORF5 in CLuTLV (covering 0.9 kb) and ORF3 to ORF6 in SBTLV (1.3 kb) [3,4]. The ORF3-encoded proteins of PMCV, GSTLV-1, and CCTLV (currently referred to as p33, p34 and p42, respectively, based on their predicted sizes in kDa) have been shown through homology searches and in silico predictions to contain a chemokine-like domain in the N-terminal end [3,7,8]. PMCV p33 is cleaved into smaller peptides upon expression, representing N-terminal and C-terminal products of variable size. However, precise cleavage sites have yet to be identified, and the reasons for the variation in product sizes are not understood [7]. The N-terminal peptides containing the chemokine-like domain are secreted, and recombinant expression of these peptides is cytotoxic to cells in culture [7]. Less is known about the C-terminal ends of p33, p34, and p42, but in silico predictions indicate the presence of a hydrophobic/transmembrane domain in all [3]. Recombinant expression of PMCV p33 variants supports a cell membrane association of this protein [7]. A chemokine-like peptide is not encoded by the additional ORFs of CLuTLV and SBTLV. However of notice, these two viruses encode small proteins, referred to as p10 and p11 (based on molecular weight), respectively, from their ORF3. These proteins are found with a pairwise sequence identity of 46% to each other, and also share a significant percentage of residues with known fusion-associated small transmembrane (FAST) proteins such as p10 from orthoreoviruses [3,4]. FAST proteins are specialized for cell–cell membrane fusion, forming syncytia. A FAST protein encoded in the SBTLV genome has experimental support, as large syncytia are observed in virus-infected cell cultures [4]. However, this feature is not yet experimentally linked specifically to the SBTLV p11 protein.

Viral fusion proteins are classified into four groups based on their structure and mechanisms of fusion. Class I-III includes fusion proteins encoded by enveloped viruses, while class IV comprises the FAST proteins, the only type of fusion protein found in non-enveloped viruses. Originally, FAST proteins were described as encoded exclusively by a few viruses within the two genera of the family *Spinareoviridae*, aquareovirus, and orthoreovirus. However, these proteins were also later described from a few species of the rotavirus genus of *Sedoviridae*, under the same *Reovirales* order [9,10,11] and also more recently from enveloped viruses of *Coronaviridae* [12,13]. FAST proteins are the smallest type of viral fusion proteins and are generally described to function at a neutral pH, they do not require a specific receptor or any other known means for fusion activation, and are the only class of viral fusion proteins specialized for cell–cell rather than virus–cell membrane fusion. Unlike enveloped viruses, non-enveloped viruses do not require fusion proteins to enter cells, but FAST proteins appear to play a role in viral replication and spread. Although not essential for virus replication, FAST proteins have been shown to enhance viral replication during the early phases of infection [14]. The mechanisms behind this are not clearly understood. Viruses with double-stranded RNA genomes replicate both strands within core particles, and it has been indicated for reoviruses that core particles within syncytia can synthesize more viral RNA than those in discrete cells due to the increased availability of substrates for viral RNA synthesis in syncytia [14]. It has also been suggested that cell–cell fusion initially promotes localized cell–cell transmission of the viruses, with subsequent enhanced virus release from apoptotic syncytia [15].

Although the majority of FAST proteins are encoded by a selection of relatively closely related viruses within *Reovirales*, they exhibit low sequence homologies. Still, FAST proteins are described by three small functional domains; an N-terminal ecto-domain, a transmembrane (TM) domain, and a C-terminal endo-domain. The ecto- and endo-domains may be characterized by combinations of structural or functional motifs like a hydrophobic patch (HP), a basic amino acid motif (polybasic, PB), a motif enriched in arginine, proline, and histidine (RPH), and a polyproline motif (PP), in addition to palmitoylated dicysteines or N-terminal myristoylation. Their lengths may vary from ~100–200 residues, and the presence and position of some of the motifs mentioned above also vary [10,16,17]).

Here we present detailed protein sequence characteristics and phylogenetic analyses of putative FAST proteins encoded by ORF3 of CLuTLV and SBTLV and compare these with corresponding characteristics of the C-terminal parts of the larger ORF3-encoded proteins from PMCV, GSTLV-1, and CCTLV-1. The results suggest that FAST proteins may also be expressed from pistolviruses. We also discuss how this finding may be supported by in vivo and in vitro observations.

## 2. Materials and Methods

### 2.1. Protein Sequence Analyses

Putative FAST protein sequences from the four members of *Pistolviridae*, CLuTLV, PMCV, GSTLV-1, and CCTLV-1, and the tentative member SBTLV, were obtained from GenBank (acc. nos. QYI86730, YP_004581251, QXI66640, and QXJ19455, respectively). The p10 protein sequence from avian reovirus (ARV) strain 176 (GenBank acc. no. AAF45151) was used for comparison. For CLuTLV and SBTLV, the sequences represent the putative FAST protein only, referred to as p10 and p11, respectively. However, the ORF3 sequences from PMCV, GSTLV-1, and CCTLV-1 each encode larger proteins, i.e., p33, p34, and p42, respectively. Based on previous experimental studies on PMCV p33 and sequence homology and similar sequence characteristics between p33, p34, and p42, only approximately the C-terminal halves of these proteins were relevant for comparative purposes in the present study. Hence, to set a reasonable limitation on the number of upstream C-terminal residues to be included, SBTLV p11 was used as the reference. This protein contains 23 amino acid residues preceding the first cysteine in a predicted discysteine noose identified in all the putative FAST proteins of the present study. This number was then used as a cut-off for the number of N-terminal residues to be included upstream of the predicted dicysteine nooses of the extracted protein sequences for the PMCV, GSTLV-1, and CCTLV-1 putative FAST protein candidates. For PMCV p33, C-terminal residues 138–302 were included, and similar residues 93–314 for GSTLV-1 p34 and residues 91–375 for CCTLV p42.

Multiple sequence alignments and additional analyses were performed using CLC Main workbench 25.0 (Qiagen, Aarhus, Denmark) and the alignments were additionally verified using the Constraint-based Multiple Alignment Tool (COBALT; NCBI). Minor manual post-alignment adjustments were also performed.

Transmembrane regions have previously been predicted for the four pistolviruses using TMpred [3,18]. As TMpred is not publicly available at present, the predictions were repeated, with additional analyses of the SBTLV p11 sequence using DeepTMHMM version 1.0.42 [19] (https://dtu.biolib.com/DeepTMHMM, accessed on 7 January 2025).

The Compute pI/Mw tool (Expasy, https://web.expasy.org/compute_pi/, accessed on 7 January 2025) was used to calculate the molecular weights of the proteins from their amino acid sequence.

### 2.2. Phylogenetic Analyses

Multiple sequence alignments for phylogenetic analyses were performed using MUSCLE implemented in MEGA X version 10.1.7 [20]. As for the alignments described in the previous section, CLuTLV p10 and SBTLV p11 were included as full-length proteins, while for PMCV, GSTLV-1, and CCTLV-1, the C-terminal parts p33^aa138–302^, p34^aa93–314,^ and p42^aa91–375,^ as defined above, were used, respectively. Additional protein sequences from a representative selection of previously published FAST proteins were included for comparisons. GenBank accession numbers for all sequences used in the analysis are given in the Results section describing the results legend. A phylogenetic tree was generated with the same software using maximum likelihood (ML) and the JTT+G model of amino acid substitution, the most appropriate model suggested by the software. Bootstrap values were calculated from 1000 replicates and values above 70 should be considered significant [21,22].

### 2.3. Sample Origin and Tissue Preparation for Histological Analyses of Atlantic Salmon Heart Tissue

Histological sections of Atlantic salmon heart tissue were available from an experimental challenge with PMCV and from a field clinical outbreak of cardiomyopathy syndrome (CMS). The material from experimentally challenged fish originated from a previous unpublished study in collaboration with external partners at conditions as earlier described [23]. In short, a heart homogenate originating from heart tissue of three individuals in a clinical outbreak of CMS was prepared as earlier described [23]. All tissue included were determined by RT-qPCR analyses to have a high load of PMCV and no detectable presence of the ubiquitous piscine *orthoreovirus*, which causes heart and skeletal muscle inflammation (HSMI) in salmon. The three tissue pieces were homogenized in Leibovitz’s L-15 medium (Gibco, Thermo Fisher Scientific, Oslo, Norway) w/ 100 μg/mL gentamycin. Experimental challenge of naïve Atlantic salmon of 150 g size (AquaGen breed) was performed using intraperitoneal injection of 0.1 mL of the filtered heart homogenate per fish under anesthesia [Tricaine Pharmaq^®^ (tricaine mesylate, “MS222”), Pharmaq, Oslo, Norway]. Fish were sampled at 4, 6, 8, and 12 weeks post-challenge. The fish were anesthetized with MS222 at sampling and killed by a blow to their head before dissection. The challenge experiment was approved by Forsøksdyrutvalget [The Norwegian Animal Research Authority (NARA)].

Additionally, organ samples were available from an internal field sample depository, which originated from a clinical outbreak of CMS in Atlantic salmon at a seawater site in Nordland County, sampled in 2011 [23].

The heart tissue samples for histology included both atrium and ventricular tissue and were fixed in 10% phosphate-buffered formalin. Infection with PMCV was confirmed by RT-qPCR analysis on RNA from parallel samples from ventricular heart tissue for both experimental challenge and the field samples [23]. Formalin-fixed tissues were processed for histological examination including sectioning at 3–4 µm and staining with hematoxylin and eosin following standard procedures. Slides were scanned and examined using NDP.view2 (Hamamatsu, Herrsching am Ammersee, Germany). A CMS diagnosis was set on the individual tissue sections according to standard CMS characteristic lesions [5].

## 3. Results

### 3.1. Pistolviruses Encode Proteins with Sequence Features Characteristic of FAST Proteins

**Figure 1:**
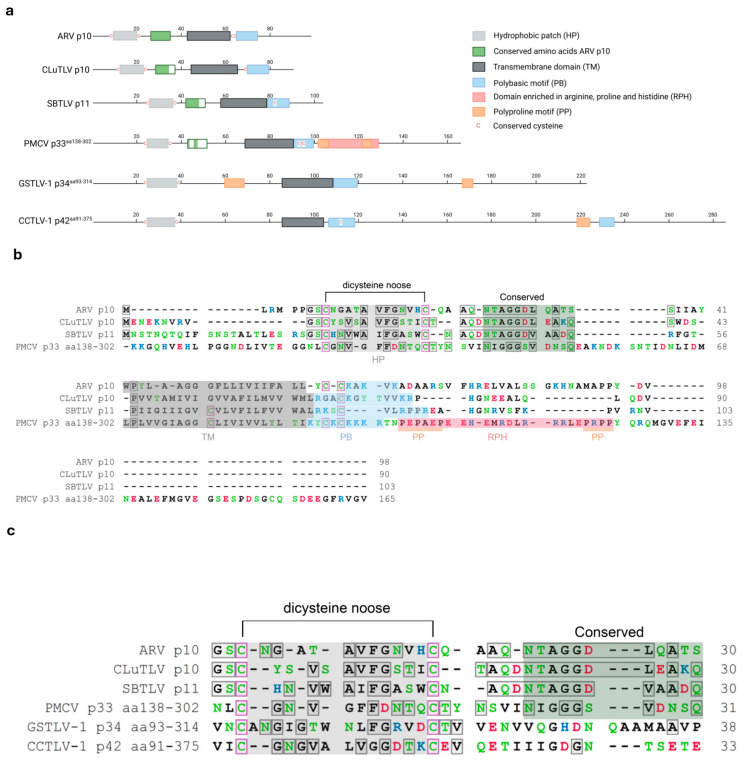
Predicted motifs and domains and conserved amino acid residues found in pistolvirus putative FAST protein sequences compared with sequence characteristics described for the ARV p10 FAST protein. (**a**) Schematic overview of the protein sequences drawn to scale. The predicted N-terminal ecto-domain, transmembrane domain, C-terminal endo-domain, and characteristic motifs are indicated, as previously described by Boutilier and Duncan [16]. Cysteines characterized as conserved are indicated specifically. Figure created in BioRender. Aase B. Mikalsen. (2025). (**b**) Protein sequence alignment of ARV p10, CLuTLV p10, SBTLV p11, and PMCV p33^aa138–302^ with conserved residues between any pair of sequences in gray shaded boxes and all cysteines in pink shaded boxes. Characteristic domains and motifs are also indicated with a shaded background as used in (**a**). (**c**) Detailed protein sequence alignment of the HP-motif with dicysteines, TM domain and PB motif of PMCV p33^aa138–302^, GSTLV-1 p34^aa93–314^ and CCTLV p42^aa91–375^. Conserved residues and cysteines are highlighted as in (**b**). Residues in (**b**,**c**) are colored according to the physiochemical properties of their amino acid side chains; black—hydrophobic, blue—basic/polar, red—acidic/polar, and green—neutral/polar. Residues are counted from the first residue (ARV p10, CLuTLV p10, and SBTLV p11) and from the first residue included for PMCV p33^aa138–302^, GSTLV-1 p34^aa96–314,^ and CCTLV-1 p42^aa94–375^ (e.g., for PMCV p33^aa138–302^, residues counted as number 1, equals residue 138 in full-length p33).

Previous analyses of the CLuTLV p10 and SBTLV p11 proteins have indicated some homology with avian orthoreovirus p10 FAST proteins [3,4]. Through more detailed comparative analyses of the protein sequences to avian reovirus (ARV) p10, we now demonstrate that the similarity is associated explicitly with key residues, motifs, and domains characteristic of orthoreovirus p10 and FAST proteins in general (Figure 1a,b). Notably, this includes two conserved cysteines previously described in orthoreovirus p10, which form an intramolecular disulfide bond, creating a noose-like fold. This structural motif comprises 11 residues, of which several are hydrophobic and form a hydrophobic patch (HP) essential for membrane fusion activity [24].

Similarly, two cysteines separated by 11 amino acid residues are present in both CLuTLV p10 and SBTLV p11, accompanied by several hydrophobic residues, suggesting the presence of a comparable HP. Two residues preceding the first cysteine and a few hydrophobic residues in HP are conserved (Figure 1a,b).

We have previously shown that the CLuTLV p10 contains a stretch of eight conserved residues of 11 conserved in the p10 of various avian orthoreoviruses [3]. The first six residues of the eight conserved between CLuTLV p10 and ARV p10 are also found in SBTLV p11 (Figure 1b). Additional nearby residues are also conserved between CLuTLV p10 and SBTLV p11. As with all FAST proteins, a putative transmembrane (TM) domain characterized by a high concentration of hydrophobic residu is located further downstream. Also comparable to the ARV p10 endo-domain is the presence of a juxamembrane motif consisting of several basic residues (the polybasic (PB) motif) and one of two conserved cysteines, which in the event of fusion activity in ARV p10 are palmitoylated [25] (Figure 1a,b). As this indicates that FAST proteins are putatively expressed from the genomes of two viruses assigned to or expected to be assigned to *Pistolviridae*, we suggested a hypothesis that all pistolviruses could have this feature. This was also supported by our previous findings describing that PMCV p33 protein is proteolytically processed into smaller peptides, of which the C-terminal peptide part is predicted to have a transmembrane domain [7].

Previous sequence homology searches on full-length PMCV p33 [3,8] and our present multiple sequence alignments including the p33 C-terminal residues 138–302 (p33^aa138–302^), did not reveal significant homology to any known FAST protein variant. However, based on the above hypothesis, we performed more detailed analyses of the p33^aa138–302^ protein sequences, which revealed the presence of conserved functionally important residues/motifs and domains characteristic of ARV p10 are present (Figure 1a). This also includes a few residues conserved between ARV p10 and/or CLuTLV p10 and SBTLV p11. More specifically, this includes the dicysteines flanking an HP, consistent with the formation of a noose with hydrophobic properties; however, for p33^aa138–302,^ the cysteines were separated by 10 residues rather than the 11 seen for ARV p10, CLuTLV p10, and SBTLV p11. Four of the eleven residues following the dicysteines/HP that are conserved in the p10 of various avian orthoreoviruses, a putative transmembrane (TM) domain characterized by a high concentration of hydrophobic residues, and the PB motif including cysteines, are also found in p33^aa138–302^ (Figure 1a,b). The predicted endo-domain of p33^aa138–302^ is longer than that of ARV p10, CLuTLV p10, and SBTLV p11, and the sequence region immediately downstream of the predicted PB motif is followed by a motif enriched in arginine, proline, and histidine (RPH) (Figure 1a,b). The extreme ends of this RPH have an increased number of prolines, which may be regarded as two separate polyproline (PP) motifs. RPH and PP motifs are not found in p10 FAST proteins, but are characteristic motifs found in the p22 (aquareoviruses) and p14/p15 (non-avian orthoreoviruses) FAST proteins, respectively [16].

PMCV shares the genomic characteristic of encoding a relatively large protein from an ORF3 at its 3′ end with GSTLV-1 and CCTLV-1, which encode p34 and p42, respectively. Despite low pairwise amino acid sequence identities among the three, common features are a predicted N-terminal signal sequence followed by a chemokine-like domain and a TM domain characterized by a relatively high number of hydrophobic residues in the C-terminal half [3]. Interestingly, multiple sequence alignment and detailed sequence analyses of the C-terminal parts of GSTLV-1 p34 (residues 93–314, p34^aa93–314^) and CCTLV-1 p42 (residues 91–375, p42^aa91–375^) confirmed the presence of a predicted dicysteine noose consisting of 12 and 14 residues, respectively, also including the HP as described for p10 FAST proteins and the CLuTLV p10, SBTLV p11 and PMCV p33^aa138–302^ (Figure 1a,c). The putative transmembrane (TM) domain and the downstream PB motif, including cysteines, were also found (Figure 1a). However, none of the 11 conserved residues previously identified in ARV p10 were identified. An RPH motif with a similar position as in PMCV p33^aa138–302^ was not apparent in GSTLV-1 p34^aa93–314^ or CCTLV-1 p42^aa91–375^ (Figure 1a). As for p33^aa138–302^, the putative endo-domains of both p34^aa93–314^ and p42^aa91–375^ are longer than those of ARV p10, CLuTLV p10, and SBTLV p11. The CCTLV-1 p42^aa91–375^ has the longest endo-domain, predicted to contain over 140 additional residues compared to ARV p10. For p34^aa93–314^, putative PP motifs were found in predicted ecto- and endo-domains. A putative PP motif was also found in the endo-domain of p42^aa91–375^ followed by an additional strong PB motif (Figure 1a).

### 3.2. Phylogenetic Analysis of Putative FAST Protein Sequences from Pistolviruses with FAST Proteins from Selected Orthoreoviruses, Aquareoviruses, Rotaviruses, and Coronaviruses

**Figure 2:**
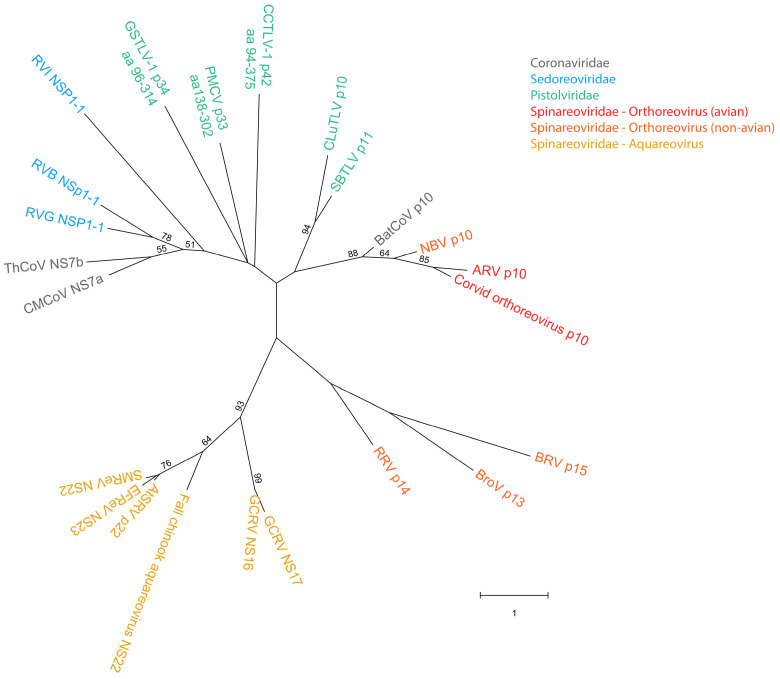
Phylogenetic analysis of pistolvirus putative FAST protein sequences with aquareo-, orthoreo-, rota- and coronavirus FAST proteins. Sequences used for CLuTLV p10 and SBTLV p11 are the full-length ORF3-encoded proteins. Sequences used for PMCV, GSTLV-1, and CCTLV-1 are the C-terminal parts of the ORF3-encoded proteins extracted from the full-length protein resulting in p33^aa138–302^, p34^aa93–314^ and p42^aa91–375^, respectively. Branch lengths are to scale and bootstrap values were calculated from 1000 replicates and values above 50 are shown. Viruses are indicated by commonly used abbreviations for the virus name, including identification of the protein equivalent to the FAST protein or putative FAST protein. Virus families are indicated by colored text (for *Spinareoviridae* a further color-coded separation by genus and host type is included). Identification of virus abbreviations with GenBank accession numbers of sequence used is as follows: CMCoV—Common moorhen coronavirus (YP 005352886.1), ThCoV—Thrush coronavirus (ACJ12059.1), RVG—Rotavirus G (AFL91897.1), RVB—Human rotavirus B (ADF57900.1), RVI—Rotavirus I (AKA63267.1), GSTLV-1—Golden shiner toti-like virus 1 (QXI66640.1), PMCV—Piscine myocarditis virus (YP 004581251.1), CCTLV-1—Common carp toti-like virus 1 (QXJ19455.1), CLuTLV—Cyclopterus lumpus toti-like virus (QYI86730.1), SBTLV—Sea bass toti-like virus (WHL55058.1), BatCoV—Bat coronavirus (AVP25418.1), NBV—Nelson Bay orthoreovirus (AAF45157.1), ARV—Avian reovirus (AAF45151.1), Corvid orthoreovirus (QJW82760.1), BRV—Baboon orthoreovirus (YP 004769555.1), BroV—Broome reovirus (ACU68609.1), RRV—Reptilian orthoreovirus (YP 009020581.1), GCRV—Grass carp reovirus (NS17 from QVL22691.1, NS16 from AZM69452.1), Fall chinook aquareovirus (YP 009351842.1), AtSRV—Atlantic salmon reovirus (ACN38055.1), EFReV—Etheostoma fonticola aquareovirus (ANN11952.1) and SMReV—Scophthalmus maximus reovirus (ADZ31982.1).

A phylogenetic analysis that included the study protein sequences from pistolviruses and the putative pistolvirus member SBTLV, and selected FAST protein sequences from aquareo-, orthoreo-, rota- and coronaviruses was performed (Figure 2). The analysis confirms as previously shown, that FAST proteins from aquareoviruses group separately from all other known FAST proteins with high bootstrap support [17]. Also, within the orthoreovirus genera, only the avian and bat p10 proteins group together with high bootstrap support, and similar support is also observed for the grouping of FAST proteins from Rotavirus species B (RVB) and G (RVG), but not I (RVI) (Figure 2).

Among the pistolvirus putative FAST protein, only sequences from CLuTLV and SBTLV group together with high bootstrap support, suggesting a more recent common precursor. Both these sequences group with orthoreovirus p10 of avian and bat origin, and although bootstrap support is low, the grouping is supported by the results in the previous section showing that important functional motifs in p10 are conserved in the two putative FAST proteins from CLuTLV and SBTLV.

### 3.3. Multinucleated Cells Indicative of Cell Fusion Are Found Correlated with SBTLV Infection In Vitro and PMCV In Vivo, Supporting Presence of Functional FAST Proteins

**Figure 3:**
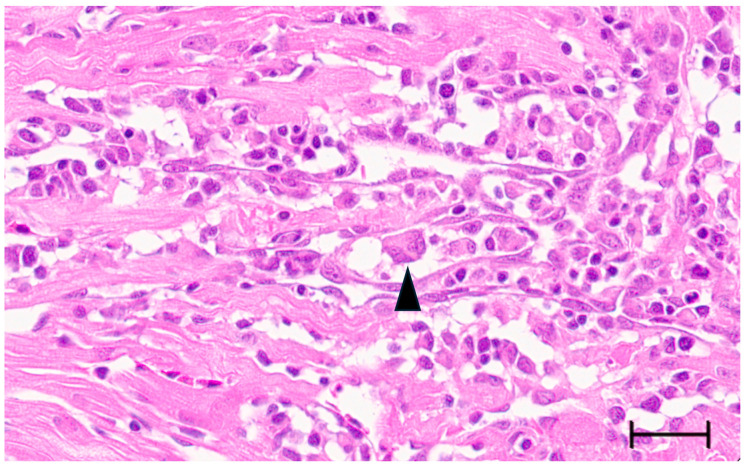
Ventricular heart tissue from Atlantic salmon experimentally challenged with PMCV with typical CMS histopathology also including multinucleated structures which may be compatible with syncytia formation (arrowhead). Bar 30 µm.

**Figure 4:**
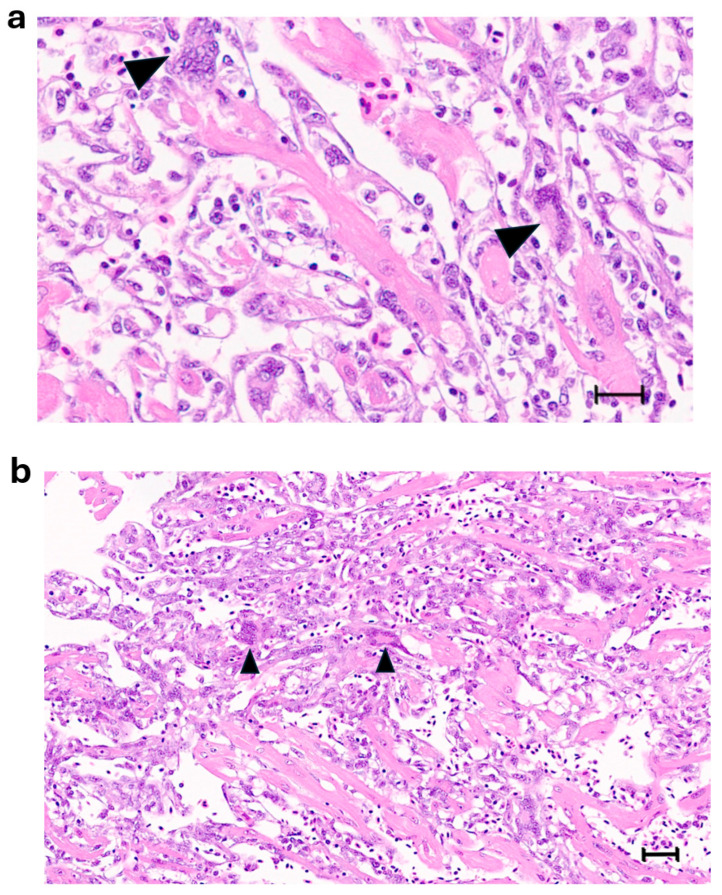
(**a**,**b**) Ventricular heart tissue from Atlantic salmon sampled from a clinical field case of CMS. Typical CMS histopathology is seen, with examples of multinucleated cells, which may be compatible with syncytia formation (arrow/arrowheads). (**a**) bar = 25 µm, (**b**) bar = 50 µm.

Large syncytia are observed in SBTLV-infected cell cultures [4] (Louboutin et al. 2023), which supports a functional FAST protein. Similar studies are not possible for the remaining pistolviruses due to a lack of cell cultures supporting viral infection. PMCV is the only pistolvirus known to cause disease in its host. To investigate whether PMCV infection induces syncytium formation and whether this phenomenon occurs concomitant with the characteristic cardiac lesions of cardiomyopathy syndrome (CMS), we examined histological sections of ventricular heart tissue from both a clinical field case and an experimental infection. Prior to histological evaluation, the presence of PMCV was confirmed by RT-qPCR detection of PMCV-specific RNA in parallel tissue samples. The analyses revealed cells of varying sizes containing multiple nuclei, often arranged in clusters or nest-like formations, which may be compatible with syncytia formation following cell membrane fusion. These multinucleated structures were observed together with characteristic CMS lesions, i.e., necrotizing myocarditis with a mix of lymphocytes and macrophages infiltrating areas of necrotic cardiomyocytes (Figure 3 and Figure 4).

## 4. Discussion

Over the past two decades, several novel viruses have been discovered that share genetic similarities with members of the former *Totiviridae* family (now reclassified into *Pseudototiviridae*, *Orthototiviridae*, and *Giardiaviridae*). The novel viruses have been assigned to distinct, newly established families, such as the *Pistolviridae* and *Artiviridae*. The well-characterized viruses of the reclassified families predominantly infect unicellular hosts and, with the exception of giardiavirus, are generally transmitted intracellularly through cell division, sporogenesis, or cell fusion rather than through extracellular release [1]. The genetic similarities between these established viruses and the newly identified ones are primarily observed in the capsid and RdRp genes and their encoded proteins, as well as in the overall genomic organization of these two core genes. However, despite these shared features, the novel viruses differ significantly by containing additional coding sequences at either the 5′ or 3′ genomic end. Furthermore, they infect more complex, multicellular hosts, including arthropod and piscine species, and are known or presumed to be transmitted via extracellular pathways. The additional coding sequences have been suggested to play a role in mechanisms related to cell entry and exit [8,26,27,28,29].

Previous studies have indicated that CLuTLV and SBTLV encode protein sequences with some sequence homology to the orthoreovirus p10 FAST proteins [3,4]. In the case of SBTLV, the expression of a fusogenic protein was further supported by observation of large syncytia in infected cell cultures. However, this phenomenon has not been explicitly linked to the putative FAST p11 protein encoded by its ORF3. Here, we provide a more detailed analysis demonstrating that, despite minimal sequence similarity, both CLuTLV p10 and SBTLV p11 possess nearly all the sequence features characteristic of p10 FAST proteins [10,17]. Notably, the putative FAST proteins of both viruses contain conserved dicysteine residues flanking an 11-residue segment with hallmark characteristics of a hydrophobic patch (HP) motif. A substantial proportion of residues conserved among avian orthoreovirus p10 variants are also preserved in CLuTLV p10 and SBTLV p11. These conserved motifs support the hypothesis that CLuTLV p10 and SBTLV p11 function as fusogenic proteins mediating membrane fusion.

As our previous studies have demonstrated, the additional ORF3 at the 3′ end of the PMCV genome encodes the multifunctional protein p33, which contains distinct sequence regions with both sequence and/or predicted structural similarities to chemokines (N-terminal region), and transmembrane proteins with similarities that could warrant classification as viroporins (C-terminal region). The full-length p33 protein is hypothesized to be anchored to the endoplasmic reticulum (ER) membrane and transported through the ER–Golgi pathway. It is cleaved at multiple sites, leading to the extracellular release of the N-terminal chemokine-like domain. In contrast, experimental data indicate that the C-terminal part remains membrane-associated [7]. Here, we provide additional evidence from protein sequence analyses suggesting that the C-terminal region of p33 (p33^aa138–302^) may share functional characteristics with FAST proteins. This is based on conserved characteristic motifs similar to those identified in CLuTLV p10 and SBTLV p11 from comparisons with ARV p10. However, the number of specific amino acids conserved in the PMCV p33^aa138–302^ is lower than in its CLuTLV p10 and SBTLV p11 protein counterparts.

The expression of a fusogenic protein from PMCV is supported by the descriptions of cells of varying size with multiple nuclei in PMCV-infected Atlantic salmon heart tissue in this study, observed as clusters or nest-like appearances of nuclei. Multinucleated giant cells and aggregates of multiple nuclei have been previously described in the heart tissue of CMS fish, observed in tissues with severe lesions [30,31,32]. This was earlier suggested to result from myocyte regeneration, and a possible link to the expression of a fusogenic PMCV protein needs experimental confirmation.

Members of the *Pistolviridae* family share key genomic characteristics and exhibit relatively high genetic homology (52–77% identity) in their capsid and RdRp genes [3]. Notably, PMCV, GSTLV-1, and CCTLV-1 all possess a relatively long ORF3 encoding p33, p34, and p42, respectively. However, it has not been documented whether GSTLV-1 p34 and CCTLV-1 p42 undergo cleavage into N-terminal and C-terminal peptides, as documented for PMCV p33 [7]. Our previous protein sequence analyses on full-length proteins suggest functional similarities among these three proteins [3] and our present sequence analyses demonstrate that the C-terminal parts of p34 and p42 also exhibit features characteristic of FAST proteins. In contrast to CLuTLV p10 and SBTLV p11, PMCV p33^aa138–302^ deviates by lacking more of the conserved key sequence residues and also deviates from the general size common for a p10-like FAST protein. Such deviations, also including the lack of motifs characteristic of p10 FAST proteins and the presence of other FAST protein motifs (like RPH and/or PP) not found in p10, are also observed in GSTLV-1 p34^aa93–314^ and CCTLV and p42^aa91–375^ to an increasing degree, respectively. Both avian and bat orthoreovirus p10 contain an HP motif of 11 residues between the dicysteines, however the analogous predicted HP motifs in p33^aa138–302^, p34^aa93–314^, and p42^aa91–375^ consist of 10, 14, and 12 residues, respectively. For ARV p10, single amino acid substitution experiments targeting the dicysteine positions, and the length and amino acid composition of the HP motif, have demonstrated that intramolecular disulfide bond formation and fusion activity are highly sensitive to the number of residues between the cysteines, and the relative number of hydrophobic residues within the HP motif [16,24]. Also, in comparison with ARV p10 and the putative FAST proteins of CLuTLV and SBTLV, p33^aa138–302^, p34^aa93–314^, and p42^aa91–375^ are predicted to possess significantly larger endo-domains. These include putative FAST protein-associated motifs, such as RPH or PP motifs, which are absent in ARV p10 but are characteristic of other FAST proteins [33,34]. Such deviations in characteristics or the general presence of motifs described as important for fusogenicity among the well-characterized FAST proteins may influence the fusion ability or efficiency of the studied pistolvirus proteins. However, the various FAST protein groups are all described to possess distinct repertoires and arrangements of important motifs and domains, yet remain fusogenic provided that the essential functional requirements for fusion initiation and execution are met. In contrast to the large virus-structural fusogens of enveloped viruses, which feature extensive ectodomains that undergo major conformational rearrangements to drive membrane apposition and fusion through the release of free energy [35], FAST proteins are small and rely on coordinated contributions from motifs and domains distributed across their full ecto-, transmembrane, and endo-domains. Among these are post-translationally modified residues that increase hydrophobicity and are critical for fusion, such as palmitoylation of membrane-proximal dicysteine motifs in the endo-domains of p10 proteins and N-terminal myristoylation in other FAST proteins. The transmembrane domain represents another example of functional conservation, despite low sequence homology among FAST protein variants; this domain has been suggested to adopt a conserved funnel-shaped architecture that is important for fusogenic activity [36]. Furthermore, studies employing chimeric FAST proteins composed of interchanged motifs and domains from p10, p14, and p15 have demonstrated that such chimeras in many cases can retain their fusogenic property. In other cases, such chimeras may exhibit a reduced or abolished fusion activity, indicating that specific domain presence, co-interactions or configurations are of importance for optimal function [16]. Collectively, these observations support a hypothesis that FAST proteins have evolved as modular fusogens, and that conservation of functional determinants over primary amino acid sequences appears to be the key requirement for FAST protein activity.

Further experimental investigations will be required to confirm fusogenic activity of the pistolvirus proteins and, if present, determine whether the variations in the dicysteine/HP motif, presence or absence of other characteristic motifs and general length of the endo-domains influence membrane fusion efficiency.

FAST proteins are commonly referred to as membrane fusogenic with the resulting outcome of syncytium formation. However, these newly identified viral proteins for which we have identified sequence features corresponding to FAST proteins, may not necessarily induce an observable syncytium formation due to functional–structural differences that affect fusogenic efficiency. One example is FAST-like proteins encoded by long terminal repeat (LTR) retrotransposons in *Drosophila melanogaster*, which have been shown to promote the formation of membrane protrusions that connect somatic cells to oocytes, rather than inducing classical syncytia [37]. The generally low sequence homology among FAST proteins is well documented, not only between proteins from different virus families, but also among members within the same genus [10,16,17,38]. Despite this sequence diversity, FAST proteins share a few essential functional features, such as a transmembrane domain directing a N_exo_/C_endo_ type I membrane topology, and an ecto-domain containing amphipathic residues and/or acylation sites for fatty acids, which are critical for mediating fusion with neighboring cell membranes. Beyond these conserved elements, FAST proteins represent a highly heterogeneous group with considerable structural and functional variation.

In addition to low sequence homologies observed between all the FAST proteins, there are also differences in the coding strategies utilized by the viruses to express these proteins. The ortho- and aquareoviruses, and the rotaviruses, are all viruses with segmented genomes and encode their FAST proteins from one of two or three partially overlapping ORFs in the specific double-stranded RNA genome segment (reviewed in [38]). In contrast, the three known coronavirus FAST proteins are encoded by separate ORFs located near the 3′ end of their non-segmented single-stranded RNA genomes. All three bat coronavirus p10s are assumed to have been acquired through recombination, thus representing recombination events that have occurred across virus families, i.e., between a single-stranded, positive-sense RNA virus and a segmented double-stranded RNA virus [12,13]. Pistolviruses (non-segmented double-stranded RNA genome) also utilize different coding strategies with the putative FAST proteins either being encoded by a separate ORF, as for CluTLV and SBTLV, or expressed as part of a larger polyprotein from which the putative FAST protein are suggested to be released upon proteolytic cleavage [3,4,7]. The different coding strategies employed to express the FAST and putative FAST proteins do not provide any immediate insight into the potential evolutionary linkage to or between the pistolviruses. However, phylogenetic analyses using RdRp genes have shown that all pistolviruses branch out in a separate cluster with very high bootstrap support within *Ghabrivirales* [1]. Previous evolutionary studies have suggested that all reovirus FAST proteins stem from at least two separate gain-of-function events giving rise to a clade consisting of the orthoreovirus p10 proteins of avian origin, a clade that includes the BroV p13, RRV p14 and BRV p15, and a clade consisting of the aquareovirus FAST proteins (reviewed in [17,38]). Also, the involvement of convergent evolution, wholly or partially, cannot be excluded.

By comparison with the simple viruses infecting unicellular hosts by intracellular spread, it is reasonable to assume that viruses infecting more advanced multicellular organisms would benefit from accessory proteins facilitating replication and spread. In viruses of the order *Reovirales*, fusogenic proteins are only found in certain species, which may suggest that membrane fusion and syncytium formation are accessory but not essential, biological properties of non-enveloped viruses [10]. However, compared to reoviruses, which possess a complex double-layered particle structure, pistolviruses are presumed to have a simpler architecture, likely composed of multimers of a single capsid protein. An accessory protein with fusogenic properties could facilitate intracellular spread, completing extracellular transmission. A similar transmission strategy has also been proposed for piscine nackednaviruses. These viruses are related to the enveloped hepadnaviruses but are characterized as non-enveloped. Several of the nackednaviruses have recently been described with a small ORF found to encode proteins with sequence characteristics corresponding to FAST proteins, suggested to be involved in cell-to-cell transmission [37,39]. Additionally, viral replication within syncytia may offer a localized environment conducive to replication, as essential gene expression and cell metabolic functions are preserved. When syncytia rupture, this strategy may enable an efficient release of high amounts of virus particles.

In conclusion, our findings provide additional detailed support to previous studies indicating that CLuTLV and SBTLV encode protein sequences with close similarity to orthoreovirus p10 FAST proteins. The presence of a functional FAST protein is further supported by our observation that protein sequences exhibiting characteristics of FAST proteins are consistently found in *all* currently known pistolviruses, indicating a required role in the viral replication cycle. These proteins may be associated with infection and transmission mechanisms that pistolviruses require for replication in more complex organisms, in contrast to their close relatives, such as members of *Totiviridae*, which replicate in single-celled hosts and rely exclusively on intracellular transmission. Expression of a fusogenic protein in these viruses is also supported by the formation of large syncytia in SBTLV-infected cell cultures and by the findings of multinucleated structures associated with CMS-characteristic lesions in PMCV-positive salmon heart tissue. However, this phenomenon has not yet been directly linked to the putative FAST proteins encoded by these viruses and hence warrants further investigations.

## Data Availability

All nucleotide and protein sequence data used in the analyses are available in NCBI GenBank as given in text. Other data related to analyses and results presented in this study are available on request, from the corresponding author.

## Abbreviations

The following abbreviations are used in this manuscript:

ARV	Avian reovirus
CCTLV-1	Common carp toti-like virus 1
CLuTLV	Cyclopterus lumpus toti-like virus
CMS	Cardiomyopathy syndrome
FAST	Fusion-associated small transmembrane proteins
GSTLV-1	Golden shiner toti-like virus 1
HP	Hydrophobic patch
kDa	Kilodalton
ML	Maximum likelihood
ORF	Open reading frame
PB	Polybasic motif
PMCV	Piscine myocarditis virus
PP	Polyproline motif
RdRp	RNA-dependent RNA polymerase
RPH	Motif enriched in arginine, proline and histidine
SBTLV	Sea bass toti-like virus
TM	Transmembrane domain
